# The Treatment of Appendicitis

**Published:** 1900-01-27

**Authors:** 


### The Treatment of Appendicitis.

It is to be hoped that the discussion on the treatment of appendicitis, which was commenced last Monday at the Medical Society of London and will be continued at a future meeting, will help to clear up some of the questions which are still in dispute in regard to that most interesting and important disease. There are few, if any, internal diseases as to the pathology of which we know so much, or as to the morbid anatomy of which in its earlier stages it has been possible to make such complete investigation as we have been enabled to make in regard to appendicitis, owing to the frequency with which the morbid specimens have been removed during life. Yet how great the differences of opinion which still remain as to the treatment of the disease. Experience on the one side says that a large number of cases of appendicitis, or at least of cases which by no known criterion can be distinguished from that condition, get well under mere medical treatment, while on the other hand careful and scientific investigations into the state of the inflamed appendix show with increasing definiteness every year how serious is the condition which underlies the manifest disease, and how grave a danger must often be entailed by leaving such inflamed and septic organs in the body. Those who advocate interference ask, and ask with great propriety, How can you expect a man to get well with such a condition within him? while the expectant physicians retort with equal force that septic or not some of these cases do get well. Thus we still remain without any sure criterion in the early stages which shall tell us whether a case should be operated on or not. Still more in doubt are we after the urgency of the case is over whether to operate or to hope that all mischief is over; and it was more especially upon this part of the question that much of the discussion on Monday turned.

The surgical aspect of the question was introduced by Mr. Lockwood, who gave an admirable demonstration of the conditions found in the appendix in the various degrees of inflammation which occur in that organ. Special interest attached to the proofs which he gave of the fact that in the mildest forms of appendicitis, those even to which some would deny the name and would rather call cases of appendicular colic, the appendix is the subject of organic disease, the mucosa being ulcerated, the basement membrane being destroyed, and a free way being left open for the extension of septic processes into the sub-peritoneal lymph spaces. Although the subject of discussion was announced as the treatment of appendicitis, Mr. Lockwood somewhat severely let it alone. His preaching was by parables and object lessons, and when he showed time after time that there is destruction of the mucosa in even the simplest cases, and that in what is spoken of as "catarrhal appendicitis," not only are the ducts crammed with microbes, but that these micro-organisms are to be found invading deeply the sub-mucous tissues, it was impossible not to see that the teaching of his parable, and the lesson driven home by his examples, was to the effect that appendicitis betokens a condition which is a constant danger to the patient, and that an appendix which has been inflamed should be removed.

Dr. Allchin, who introduced the subject of the medical treatment of appendicitis, was equally clear that many cases, indistinguishable from others which ended in suppuration, ultimately not only got well but remained well, and although he quite concurred in the necessity for surgical intervention in certain cases, he was equally positive that there were many in which it was unnecessary. Thus, between the positive teaching of experience, as detailed by Dr. Allchin, and the equally positive teaching of scientific investigation, as represented by Mr. Lockwood's admirable specimens, we have an almost absolute divergence, and the plain man may well ask how can such differences between honest observers be explained? Perhaps a clue to the answer may be found in the very apposite remarks made by Mr. Barker as to the microcidal and self-protective power of the peritoneum. The whole force of the argument in favour of early operation in appendicitis and the more frequent removal of the appendix after the inflammation has apparently subsided, hinges on the assumption that a septic appendix must do harm; and the real point of Mr. Lockwood's demonstration was that it showed how many even of the simple cases are really septic, and so far as power for evil is concerned, must be considered along with the rest. Mr. Barker, however, urged that the fact of an organ being infected with septic germs was by no means of necessity a reason for removing it. We must, he said, give the peritoneum its due. If the peritoneum were not able to take care of itself to a large extent, such operations as were constantly being done for the removal of the results of septic infection within its cavity would be out of the question. But, in fact, even when suppuration was known to be widespread, he did not attempt to wash out the whole abdomen—partly because he knew it to be impossible. Surgeons seemed to think that if there was septic mischief it would spread; but that was by no means always the case. Indeed, the very arguments which would lead to the removal of every septic appendix should lead also to much wider removal of tissue, especially of omentum, than was ever done.

Thus, perhaps, may we understand how it is that, as is maintained by the expectants, such cases sometimes get well; and, however septic they may be at the time, ultimately lose their power for evil. But which cases? The rough rule of removing every diseased appendix, which is being advocated in all its baldness in America, has at least simplicity in its favour; and if it is to be withstood, we must ask the physicians to show us how to discriminate between those that must be operated on and those that may safely be left in their hands.

